# Skin cancer in solid organ transplant recipients: are mTOR inhibitors a game changer?

**DOI:** 10.1186/s13737-014-0022-4

**Published:** 2015-01-14

**Authors:** Edward K Geissler

**Affiliations:** Section of Experimental Surgery, Department of Surgery, University Hospital Regensburg, Franz-Josef-Strauss-Allee 11, Regensburg, 93053 Germany

**Keywords:** mTOR, Organ transplantation, Post-transplant malignancy, Calcineurin inhibitors, Skin cancer

## Abstract

While immunosuppressive agents are necessary to prevent the rejection of transplanted organs, and are a great medical success story for protecting against early allograft loss, graft and patient survival over the long term are diminished by side effects from these same drugs. One striking long-term side effect is a high rate of skin cancer development. The skin cancers that develop in transplant recipients tend to be numerous, as well as particularly aggressive, and are therefore a major contributor to morbidity and mortality in transplant recipients. An apparent reason for the high incidence of skin cancer likely relates to suppression of immune surveillance mechanisms, but other more direct effects of certain immunosuppressive drugs are also bound to contribute to cancers of UV-exposed skin. However, over the past few years, evidence has emerged to suggest that one class of immunosuppressants, mammalian target of rapamycin (mTOR) inhibitors, could potentially inhibit skin tumour formation through a number of mechanisms that are still being studied intensively today. Therefore, in light of the high skin cancer incidence in transplant recipients, it follows that clinical trials have been conducted to determine if mTOR inhibitors can significantly reduce these post-transplant skin malignancies. Here, the problem of post-transplant skin cancer will be briefly reviewed, along with the possible mechanisms contributing to this problem, followed by an overview of the relevant clinical trial results using mTOR inhibitors.

## Skin cancer in transplant recipients

Key breakthroughs in the discovery of immunosuppressive agents over the past three to four decades have expanded the routine use of solid organ transplantation to the point where demand for organs far exceed their availability. Organ transplantation has unquestionably been a success story brought about by medical research. In the current state of this success, however, the transplantation community has come to the realisation that while early organ allograft and patient survival are excellent, long-term outcomes have not improved substantially over the past couple of decades. The inadequacy of organ availability has heightened this realisation of unimproved long-term outcomes and has caused transplant organisations to search for reasons of explanation. There are indeed a number of factors responsible for poor long-term results, including chronic allograft rejection, cardiovascular disease, infectious complications and development of malignancies [1,2]. Regarding the latter, it is particularly difficult to accept the complication of post-transplant malignancies since these recipients typically die with a well-functioning transplant. Adding to the frustration is the fact that the malignancies commonly occur after ten or more years post-transplantation, meaning that the recipient is otherwise adapting well to the allograft over the long term by not immunologically rejecting the organ. New strategies aimed at reducing malignancies in these patients are therefore clearly needed.

By far the most common malignancies occurring in transplant recipients are skin cancers. Data from the different registries suggest that more than half of transplant recipients will experience at least one of a variety of skin malignancies [3]. The predominant epithelial skin cancer in this population is cutaneous squamous cell carcinoma (SCC), which is characterised by lesions that subsequently increase in frequency and multiplicity after the first lesion is diagnosed [4,5]; the occurrence rate is increased by approximately 65–100-fold compared to the general population [6]. Successful strategies to combat these highly frequent and too often aggressive tumours would have a significant impact on the quality of life for long-term allograft recipients. Other skin tumours are also increased in organ transplant recipients, although less frequently. Basal cell carcinomas (BCC) are the second most common skin tumour post-transplantation, although they do not display a more aggressive behaviour compared to the general population [7,8]. Interestingly, while BCC are more common (4:1) than SCC in the general population, this ratio is completely reversed in transplant recipients, indicating the extraordinary predilection for SCC development in these immunosuppressed people. Highly aggressive cutaneous Merkel cell carcinomas are also increased 5–10-fold in transplant recipients [9], and Kaposi’s sarcoma is dramatically increased to a rate up to 500-fold more than age-matched controls [10]. Although otherwise rather rare, Kaposi’s sarcoma accounts for approximately 6% of all malignancies in transplant recipients. Unlike SCC which tends to occur several years after transplantation, Kaposi’s sarcoma occurs within the first year or two post-transplantation and develops in close association with human herpesvirus 8 (HHV-8) reactivation. Finally, it should be mentioned that cutaneous malignant melanoma shows only a slight increase in risk for transplant recipients, but high mortality rates have been reported [11,12], making it a serious concern for these patients. A recent report indicates that cutaneous melanomas in kidney transplant recipients show a particularly aggressive tumour behaviour that is reflected in demonstrably poor outcomes [13].

## Mechanisms for increased skin cancer

While the complex topic of causes for these various types of skin cancer cannot be comprehensively covered in this review, a few mechanisms should be mentioned for establishing a basic knowledge. The most obvious factor thought to affect cancer development in transplant recipients is systemic suppression of the immune system caused by anti-rejection drugs. There are two primary consequences of immune suppression, one being the inhibition of immune reactions capable of recognizing and destroying tumour cells [14] and the other being the permissiveness of viral infections that are associated with common skin cancers (e.g. HHV-8, Kaposi’s sarcoma; human papillomavirus, SCC; Merkel cell polyomavirus, Merkel cell carcinoma) [15]. However, it is becoming increasingly clear that non-immunological factors are also important, such as direct effects of certain immunosuppressive drugs on neoplasms. For example, cyclosporine (calcineurin inhibition) is known to promote tumour cell invasiveness [16] and boost vascular endothelial growth factor-induced angiogenesis that nourishes cancer growth [17]. Importantly, cyclosporine is also known to have an inhibitory effect on DNA repair mechanisms [18], which are understandably critical for the repair of damage caused by UV light on exposed areas of the skin. Similarly, another immunosuppressant, azathioprine, is well known to be mutagenic, acting synergistically with UV radiation damage in promoting skin cancer pathogenesis [19-21]. Therefore, commonly used immunosuppressants in transplant recipients demonstrate activities that are likely to promote the development of skin tumours. Indeed, several experimental animal studies support this theory, in that treatment with daily immunosuppressive doses of cyclosporine causes tumours to grow at a faster rate [17,22,23].

## Potential for mTOR inhibition to reduce skin cancer

There has been much debate, testing and intensive continuing research regarding the potential anti-tumour effects of mammalian target of rapamycin (mTOR) inhibitors [24-26]. Inhibition of mTOR sets up a unique molecular scenario whereby it is plausible that various aspects of tumour development could be inhibited while at the same time causing a general immunosuppression that protects allografts from rejection. The mTOR pathway is essential for cell growth and proliferation and influences processes such as autophagy, serving as a pivotal regulatory point for the coordination of cell signalling with nutrient availability [27,28]. Interestingly, both cells of the immune system and of tumour entities require this type of coordination and therefore are influenced by inhibition of mTOR. Although this concise review does not allow space for going into details about the intricacies of the two different mTOR complexes, it should be mentioned here that the mTOR inhibitors used for immunosuppression in transplant recipients (sirolimus and everolimus) primarily inhibit mTOR complex 1 [29,30], and this is where the discussion will focus; subsequently in this review, when referring to mTOR inhibition, it is generally in reference to inhibition of mTOR complex 1.

In the case of the immune system, a primary effect of mTOR inhibition is through blocking of the IL-2 proliferation signal to T cells, which is necessary to expand T cell responses directed specifically against the alloantigens expressed by the transplanted organ; thus, mTOR inhibition is highly immunosuppressive. It should also be mentioned however that mTOR inhibition has other effects on specific populations of immune cells, including inhibition of antigen-presenting cell maturation [31] and promotion of T regulatory cell development [32]. The complex effects of mTOR inhibition on the immune system have been reviewed elsewhere [33], but it can be summarised here that there are both inhibitory and potent stimulatory effects on immune cell populations that are likely mediated by mTOR inhibitors. Therefore, while clearly immunosuppressive, it should be kept in mind that mTOR inhibitors have widely different effects on immune cells, making it difficult to predict what the overall effect may be on tumour formation; indeed, the effect may vary from one tumour type to the other, depending on their immunogenicity.

Nonetheless, there is a large body of literature that indicates that mTOR inhibitors may be effective against various aspects of tumour development. Indeed, there are several possible mechanisms of action that have been described. While this is not meant to be a full review of anti-tumour mechanisms potentially involved, it is worth pointing out some of the key roles of the mTOR pathway that could impact skin cancer development. For instance, angiogenesis relies on vascular endothelial growth factor expression and signalling in supportive tumour vessel structures, which are dependent on the mTOR pathway [34]. We have shown that mTOR inhibitors dramatically block tumour angiogenesis, which leads to substantially reduced tumour growth in animal models [17]. Another aspect is the multitude of mutations in key signalling molecules that have been described upstream of the mTOR complex 1 signalling node that result in constitutive activation of the mTOR pathway and thus uncontrolled cell growth and proliferation; included in this extensive list are mutations in PTEN, TSC1/2 and Ras/Raf that lead to mTOR complex 1 activation [24]. Pkd-1 mutations can also result in triggering of the mTOR pathway and have been linked to cell proliferation and the development of polycystic kidney disease; blocking mTOR with rapamycin can substantially inhibit the proliferation of cysts in mice with this conditionally expressed mutation [35]. Therefore, inhibition of mTOR has substantial mechanistic potential to be considered overall as an anti-cancer agent.

Particularly relevant for the topic of skin cancers in transplant recipients may be the recently discovered potential anti-viral effects of mTOR inhibitors [36,37]. The close association of viral infection (as discussed earlier) with the development of different skin malignancies brings this property into focus. While the mechanisms for the anti-viral effect are not well understood yet, it seems some viruses are responsive to mTOR inhibition [37], and mTOR inhibitors have been shown to boost CD8 T cell responses induced by viral vaccines, even at immunosuppressive doses in non-human primates [38]. Therefore, if the anti-viral effects of mTOR inhibitors are better characterised and eventually confirmed, this may be another important mechanism to consider in the fight against post-transplant skin malignancies.

## Key skin cancer trials in transplant recipients

Apart from the experimental and theoretical view towards reducing skin cancer in organ transplant recipients with mTOR inhibitors, the first clinical trials with this aim have now been published. In this part of the review, results from the key trials will be discussed (see Table 1).

**Table 1:** **Randomised multicentre clinical trials with skin cancer as the primary endpoint**

**Study**	**Patient number**	**Primary endpoint**	**Primary endpoint result**	**Comments**
Australian study	Control: 47 (23)^a^	Number of new NMSC/patient/year	Significant	A significant decrease in SCC, but not BCC
	mTORi: 39 (19)			
TUMORAPA	Control: 56 (12)	Survival free of new SCC	Significant	patients with 1 prior SCC benefited significantly; those with multiple previous SCC did not significantly benefit
	mTORi: 64 (22)			
RESCUE	Control: 81 (14)	Risk of new SCC	Not significant	HR improved in 1-year analysis, but did not after 2-year follow-up
	mTORi: 74 (39)			

*SCC* squamous cell carcinoma, *BCC* basal cell carcinoma, *NMSC* non-melanoma skin cancer, *Control* patients not receiving mTOR inhibitors, *mTORi* mTOR inhibitor group, *HR* hazard ratio.

^a^Number in parentheses represents patients dropping out of study. For the definition of a dropout and number of patients that completed the study according to the protocol, see the individual publications.

### Australian skin cancer trial

The first report of a randomised, multicentre clinical trial tested the effects of switching from calcineurin inhibitor-based immunosuppression to sirolimus on the risk for development of non-melanoma skin cancer (NMSC) in renal transplant recipients [39]. A total of 87 transplant patients at a high risk for NMSC were randomised at least 1 year after transplantation to continue on a calcineurin inhibitor-based immunosuppression or be switched to sirolimus; the primary endpoint was number of biopsy-confirmed new NMSC per patient per year. Over a 2-year observation period, SCC occurred at a significantly lower rate in the sirolimus group of patients, although the rate of basal cell carcinomas was the same. Also, a lower rate of new NMSC developed in the sirolimus-converted group. Furthermore, it took more than twofold the number of days for a new NMSC to occur in the sirolimus versus control group of patients. Importantly, the conversion to sirolimus did not result in an increased risk for having an acute kidney rejection episode, but there was a high rate of treatment discontinuation in the sirolimus group (42.6%) due to typical well-known side effects associated with mTOR inhibitor use. Nonetheless, the switch from calcineurin inhibitor to mTOR inhibitor did have a positive clinically significant effect on skin cancer development in this study.

### TUMORAPA

Within a couple of months after the release of the Australian trial results, Euvrard and colleagues published data from a similar clinical trial that focussed on SCC in kidney transplant recipients [40]. This trial used a combination of data from two registered trials, TUMORAPA-1 and TUMORAPA-N, which aimed to enrol patients with a first SCC and after multiple SCC post-transplantation, respectively. A total of 120 patients were enrolled in this combined study, which was a calcineurin inhibitor to sirolimus conversion protocol looking at SCC-free survival 2 years after randomisation. The rate of SCC-free survival was significantly longer with sirolimus conversion, where 22% of patients developed new SCC compared to 39% in the group maintained on calcineurin inhibitors. An important observation in this study was that significance in the mTOR inhibitor effect held in the group of patients that had only a single pre-randomisation SCC, but was lost when recipients had multiple SCC prior to entry into the study; it should be added however that the study may not have been adequately powered to see such a difference. Another potentially important observation was that patients did experience significant side effects causing treatment discontinuation in the sirolimus conversion group, but these side effects were substantially fewer when a slower conversion (over >7 days) was performed. The Australian and TUMORAPA trials in large part reached a similar conclusion that mTOR inhibitors inhibit skin cancer in high-risk kidney transplant recipients.

### RESCUE

Another related clinical trial in the Netherlands and the United Kingdom was performed during approximately the same time frame as the previously cited trials and is referred to as the RESCUE trial [41]. Similar to the first two trials discussed, RESCUE was a randomised, multicentre study with a 2-year follow-up in kidney transplant recipients that had experienced at least one pre-randomisation SCC. A total of 155 patients were randomised into a group maintained on their non-mTOR inhibitor-based regime or into an arm where conversion to sirolimus was performed. The primary endpoint was met when the patient developed a new SCC within the 2-year observation period. Results from this trial are interesting to contrast with the other two trials, since no significant decrease in new SCC (the primary endpoint) was observed in the sirolimus group as a whole after 2 years, but additional analyses revealed that there was a significant 50% decrease in risk (measured by hazard ratio) after only 1 year of observation. A provocative conclusion to this finding is that mTOR inhibitors delay, but do not prevent, skin cancers in these patients; a longer-term follow-up would be necessary to begin to address this issue. An additional finding in this study was a substantial improvement in hazard ratio for those patients that entered the trial with only one previous SCC, which is consistent with the results from TUMORAPA. As with the other trials, a high discontinuation rate (39%) was observed in the sirolimus group due to adverse side effects.

### Additional useful trial results

It should be mentioned that other trials have investigated the effect of mTOR inhibitors on development of skin cancer in transplant recipients, including a small randomised single-centre German study published by Salgo and colleagues [42]. This trial showed also that sirolimus conversion in renal transplant recipients has an inhibitory effect on development of NMSC, where only 1/16 sirolimus-converted versus 8/17 control group patients developed skin cancer. Results from large sirolimus conversion trials, where *de novo* malignancies were examined only as secondary endpoints, also show a beneficial effect in terms of skin cancer occurrence [43,44]. Evidence is emerging to show that everolimus is also likely to have a similar inhibitory effect on skin cancer in transplant recipients [45,46]. Finally, it is important to point out the success reported in the treatment of Kaposi’s sarcoma with mTOR inhibitor conversion in transplant recipients. Several key studies have shown a dramatic effect of mTOR inhibitor conversion on lesion stabilisation, reduction and even disappearance [47-49]. Kaposi’s sarcoma is potentially a good target for mTOR inhibitors, since they are highly vascular tumours and linked directly with HHV-8 viral infection.

## Conclusions

Skin cancer is a substantial problem in organ transplant recipients that is attributed to the immunosuppressive drugs that are most commonly used to prevent allograft rejection. In particular, evidence suggests that the calcineurin inhibitors and azathioprine contribute to the development of skin neoplasms both through paralysis of immune surveillance and by promotion of tumour vascularisation, cancer cell invasiveness and exacerbation of DNA damage or inhibition of DNA repair. While suppression of the immune reactivity against cancer cannot be completely avoided with an immunosuppressive drug, mTOR inhibitors have a unique potential to both suppress an immune response to the organ allograft and promote mechanisms that can potentially inhibit tumour development. The basic research to better understand this phenomenon continues to evolve at a steady pace, but in the meantime, clinical trials in transplant recipients already indicate that skin cancer can be reduced by substitution of calcineurin inhibitors with mTOR inhibitors. Results from clinical studies suggest also that patients at a high risk for skin cancer receive the greatest benefit when mTOR inhibitors are used early, before multiple lesions have already developed. The key to taking advantage of this benefit will be the institution of improved protocols that minimise the adverse side effects of mTOR inhibitor use in transplant recipients that undergo immunosuppression switching. Therefore, mTOR inhibitors could be a “game changer” to reduce the problem of post-transplantation skin cancer, but more research will be necessary to optimise clinical protocols that will be widely acceptable for high-risk patients that can benefit from this general strategy.
